# Ribotyping of *Clostridium difficile* strains associated with nosocomial transmission and relapses in a Swedish County

**DOI:** 10.1111/j.1600-0463.2012.02950.x

**Published:** 2012-07-25

**Authors:** Cecilia Magnusson, Marlene Wullt, Sture Löfgren, Peter Iveroth, Tomas Åkerlund, Andreas Matussek

**Affiliations:** 1 Department of Infectious Diseases, County Hospital RyhovJönköping, Sweden; 2 Department of Infectious Diseases, University Hospital MalmöMalmö, Sweden; 3 Clinical Microbiology Laboratory, Department of Laboratory Medicine, County Hospital RyhovJönköping, Sweden; 4 Department of Infectious Disease Control, County Hospital RyhovJönköping, Sweden; 5 Swedish Institute for Infectious Disease ControlSolna, Sweden

**Keywords:** *Clostridium difficile*, molecular epidemiology, ribotyping, transmission, CDI, hygiene measures

## Abstract

*Clostridium difficile* is an emerging threat in hospital environments. To analyse possible transmission and to distinguish between relapse and reinfection a collection of *C. difficile* isolates, sampled from 162 consecutive episodes of *C. difficile* infection, were PCR ribotyped. Two ribotypes (001 and 012) were prone to cause nosocomial acquisition. Moreover, ribotype 001 had a tendency to cause relapses as almost one in two patients with this ribotype had one or more relapses. By using PCR ribotyping strains inclined to cause relapses and strains associated with hospital transmission might be detected. This enables optimized hygiene measures and may improve the choice of treatment regimen.

*Clostridium difficile*, an anaerobe Gram-positive sporulating rod, is an emerging threat in hospital environments. The pathogenesis is based on production of enterotoxin A and/or cytotoxin B 1–3. A binary toxin, associated with a more severe disease as well as an increased rate of recurrences, has recently been detected 4. Molecular typing enables the detection of virulent strains. Moreover, typing may clarify whether a cluster of *C. difficile* infection (CDI) is due to transmission of a single strain or a more random occurrence 5, 6. It should, however, be noted that information on strain distribution in the community is required to fully explore the epidemiology of *C. difficile* in hospitals.

A patient suffering from a recurrence has an increased risk of additional recurrences. Treatment of recurrent CDI remains challenging since high quality treatment studies are lacking 7. In this study we investigated the distribution of PCR ribotypes causing CDI both in the community and in hospital settings. We identified two ribotypes (012 and 001) highly associated with nosocomial transmissions. We also found ribotyping suitable to evaluate recurrences, and identified ribotype 001 as prone to cause relapses.

## Material and Methods

### Study population and material

We reviewed all diagnosed CDI episodes (n = 360) in Jönköping County, Sweden from September 2000 through August 2001. The county has 330 000 inhabitants, and three hospitals are located in this area. Epidemiological data regarding date of illness, date and duration of hospitalization, ward, transfer between wards and hospitals, and date of sampling, were collected by reviewing medical records of the patients. We performed PCR-ribotyping on 188 available isolates from the 360 episodes.

### Definitions

CDI was defined as diarrhoea and presence of *C. difficile* toxin in faeces. An episode was considered as primary if the patient had no prior history of CDI within 6 months, otherwise it was considered as a recurrence. A primary episode starting 48 h after hospital admission or up to 60 days after discharge was considered hospital associated (HA). Otherwise it was considered as community acquired (CA). A relapse was defined as a recurrence caused by the same ribotype as the primary episode, and a reinfection was defined as a recurrence with a different ribotype. A HA episode was regarded as nosocomially acquired (NA) when the isolated ribotype had been identified in a patient at the same ward within either 2 months or 12 months previously. The two different time periods were used to evaluate the ward environment as a potential reservoir for transmission. The nosocomial acquisition rate was determined as the proportion (in %) of NA to HA episodes.

### Faecal toxin test, culture and PCR-ribotyping

Detection of *C. difficile* toxins A and B was performed using the ELISA Premier Toxins A & B (Meridian Bioscience, Cincinnati, OH, USA) according to the manufacturer's instructions. Culture of *C. difficile* was performed according to the Swedish reference method (http://www.referensmetodik.smi.se/w/Clostridium_difficile-laboratoriediagnostik). PCR amplification and analysis of PCR products were performed as previously described 8. All banding patterns were analysed by one technician blinded for epidemiological data.

## Results

## CDI episodes and ribotype distribution

A total of 360 episodes (109/100 000 inhabitants) of CDI were identified in 284 patients. In total 32 different ribotypes were identified in the 188 samples, representing 162 different episodes in 137 patients. The five most common ribotypes, in ranking order, 012, 001, 005, SE21 and 002 were recovered from 72 of the 137 (53%) patients (Table 1). Of the 162 episodes 110 (68%) were primary of which 84 (76%) and 26 (24%) were characterized as HA and CA, respectively. In primary HA episodes, 001 and 012 were the most frequent ribotypes representing 33% of these episodes. Primary episodes of 001 were all except for two HA (86%) and of 012 all except for one (94%) (Table 1).

**Table 1:** PCR ribotypes of *Clostridium difficile* isolates from patients with CDI during 1 year in Jönköping County, Sweden

Ribotype^1^	Patients (no.)	Primary episodes (no.)	CA	HA	Patients with recurrences (no.)	Total no. of episodes	NA 2 months	NA 12 months
012	20	17	1	16	7	23	13	14
001	18	14	2	12	11	27	9	10
005	12	11	2	9	3	14	5	5
SE21	12	11	4	7	3	14	1	2
002	10	9	4	5	1	10	0	1
020	8	7	0	7	2	9	0	0
081	6	4	0	4	2	6	2	2
015	6	5	3	2	2	6	0	0
023	6	6	2	4	1	8	0	2
014	6	4	1	3	4	8	2	2
SE14	4	4	2	2	0	4	0	0
078	4	3	0	3	1	5	1	1
Other (n = 20)	25	15	5	10	13	28	0	1
Total	137	110	26	84	50	162	33	40

CA = community acquired, HA = hospital associated, NA = nosocomially acquired.

1 Ribotypes are labelled according to the Cardiff PCR-ribotyping library, except for two (SE14 and SE21), which are labelled according to the Swedish library.

## Recurrences

A total of 50 patients (36%) suffered 52 recurrences and the majority of these (n = 47) occurred within 2 months (data not shown). In 29 patients isolates were available for ribotyping from both the primary infection as well as the recurrence, representing 26 relapses and 5 reinfections (Table 2). Ribotype 001 caused nine of these relapses (36%) whereas other ribotypes caused one to three (Table 2). The five reinfections were all HA and were caused by five different ribotypes.

**Table 2:** PCR ribotypes and antibiotic treatment (ab) in patients with recurrences

Pat	ab 1	Ribotype 1	ab 2	Ribotype 2	ab 3	Ribotype 3	Relapse	Reinfection	CA	HA
1	C^1^+F	001		001		001	2			1
2	C	001		001			1			1
3	F+D	001		001			1			1
4	C+D+A	001		001			1			1
5	E+B	001	F+B	001			1			1
6	B+C	001	C	001			1			1
7	A+B+F	001		001			1			1
8	B	001		001			1			1
9	A	012		012			1			1
10	A	012		012			1			1
11	B+A	012	B+E	012			1			1
12	A	005		005			1			1
13	B	005		005			1			1
14	C+F	SE21		SE21			1			1
15	E+A	SE21		SE21			1		1	
16	F	023		023		023	2		1	
17	C^1^	029		029			1			1
18	C^1^	029		029			1			1
19	B+E+F	014		014			1			1
20	B+F	014	B	014			1			1
21	B	020		020			1			1
22	F+D	078		078			1			1
23	C^1^	SE36	B	SE36			1			1
24	A	015		015			1			1
25	B+D	053		053			1			1
26	F+D+B	012	E	SE1				1		1
27	E	003		SE2		081		2	1	
28	B+C	SE19e		002				1		1
29	A	001		SE19e				1		1

A = Clindamycin, B = Cephalosporins, C = Isoxasolyl penicillin, D = Fluoroquinolones, E = Penicillin G, F = Other antibiotics and G = Unknown antibiotic, CA = community acquired, HA = hospital associated.

1 Antibiotic given as a single prophylactic dose.

## Nosocomial acquisition

Each HA episode including the five reinfections was evaluated for the possibility of NA. The NA rate within 2 months was 37% (33/89) and within 12 months was 45% (40/89) (Table 1). The NA rate among patients with ribotype 001 was 75% (9/12) and 83% (10/12) within 2 and 12 months, respectively. For ribotype 012 these figures were 81% (13/16) and 87% (14/16) within 2 and 12 months, respectively. All infections with ribotype 020 (n = 7) were HA.

## Discussion

The current interest in *C. difficile* focuses on molecular epidemiology to disclose hospital transmission and to detect strains of higher virulence, such as ribotype 027 2, 3. Although ribotype 027 was not found in this study for which samples were collected 2000–2001, we found two other ribotypes, 001 and 012 with traits of higher virulence.

The overall incidence of CDI and the major ribotype distribution in the Jönköping County, was in accordance with previous Swedish data 8 from the same time period, although we detected a higher rate of recurrences. In our investigation 36% of the patients had one or more recurrences, and among relapses a large proportion was caused by ribotype 001. In fact, 8 of 18 patients with 001 had one or more relapses. This might indeed contribute to the higher NA rate of this ribotype since seven of the eight patients with relapses were readmitted, thus, introducing this ribotype again to the hospitals (data not shown).

Of the 29 patients with recurrences no more than six received antibiotic treatment between the episodes, and only two of these six patients had ribotype 001 (Table 2). This indicates that a new antibiotic course after resolution of CDI was not required for a relapse with 001. Furthermore, we compared the antibiotic regimes administered to patients with ribotypes 001 and 012 before their primary infection. The frequency of broad spectrum antibiotics was similar in the two groups (data not shown), but we noticed a statistically significant difference in rates of relapse (p = 0.04) (Table 2).

The fact that some strains seem prone to cause relapses needs to be further investigated and molecular typing is an important prerequisite to explore this field in detail. One important future aspect to consider is how treatment regimes could be optimized to prevent relapses 7. This would decrease the burden of illness for individual patients and may also minimize nosocomial transmissions and hospital costs. The five reinfections detected were caused by five different ribotypes.

Evaluation of the hospital epidemiology of *C. difficile* should include comparison of hospital and community ribotype distributions. If such data are not available, a high frequency of a ribotype in HA as well as in NA cases, might be misinterpreted as an outbreak although it merely reflected the prevalence of the ribotype in the community. However, in our study, the low frequency of 001 and 012 in the CA episodes together with their high NA rate compared to other ribotypes indicate a higher tendency of transmission within hospitals.

The NA rates for 001 and 012 were high within the 2 months time span. Extending the time period of observation to 12 months did not increase the NA rate substantially. This indicates that the risk of NA in our study correlated to person-to-person transmission rather than exposure to endospores in the environment, which is in line with suggestions by McFarland et al. 9. In fact, most transmissions seemed to occur when patients were hospitalized at the same ward during overlapping time spans (data not shown). The NA rate observed was higher compared to figures in other Swedish counties 5, 8. This might reflect differences in antibiotic treatment policies, compliance to hygiene measures and/or the fact that we were able to trace all patient transfers between wards.

Among the 32 ribotypes found, none seemed to be transmitted in the community as they only occurred in one up to four patients (002 and SE21, respectively). The ribotypes 020, 078 and 081 were only found in HA episodes, but none of them seemed prone to transmission as no NA was detected.

In conclusion, we found ribotype 001 to be a major cause of relapsing CDI. Ribotypes 001 and 012 were prone to nosocomial transmission emphasizing the importance of molecular typing for detection of contagious and virulent strains. In future, identification of strains with enhanced virulence may help to optimize treatment and to prevent relapses.
